# Buffalo Medical Association

**Published:** 1879-09

**Authors:** 


### Society Reports.

---

### BUFFALO MEDICAL ASSOCIATION.

*Stated Meeting, July 1st, 1879.*

THE PRESIDENT, DR. LUCIEN HOWE, IN THE CHAIR.

The members present were Drs. Keene, Trowbridge, White, Bartlett, Cary, Mynter, Hauenstein, Barnes, Moody, and Fowler. Dr. Fowler acted as Secretary.

The application of Dr. Davidson for membership having been received, the Association listened to the reading of an interesting paper by Dr. Charles Cary, on the "Support of the Perineum," of which the following is a brief abstract:

The subject I have chosen to bring up for discussion this evening, has not the virtue of novelty, but is as old as scientific midwifery itself.

It has been fully written up, various views have been maintained and abandoned, but still room sufficient is left for improvement in the present management of the perineum during labor. I may go even further, and add that I consider the support as ordinarily given, useless, if not harmful, and is a procedure to which might aptly be applied the term of meddlesome midwifery. In attacking a practice which is so general as almost to be universal, I am fully aware of the opposition which will be brought forward, and it is perhaps somewhat for that reason that I have taken this subject; being fully convinced that the friendly discussion which follows such a paper is oftentimes far more beneficial to us all than the mere paper itself. In questioning some of our best practitioners in this city, I have not found one who disapproves of the ordinary method of support. And yet physicians who pretend to follow the plan proposed by Vealpeau and by Cazeaux, nevertheless, at the very moment when least demanded—at the time when pains are hard and the entire perineum is protruding, being pressed out by the head, many physicians undertake to slip the posterior fourchette over the child's head, by pushing it backwards. It is a temptation that many do not resist. They "crown" the child, as it is called, just when on the contrary every effort should be made to prevent the act.

Dr. Cary then quoted at length from the works of Ramsbotham, whose strongly favored support; from Tyler Smith, Dr. Churchill, and Dr. Meadows, who advised it only under certain circumstances or were indifferent regarding it; and from Dr. Grailey Hewitt, who says vehemently: "It is unnecessary or mischievous." Leishman, whose views are generally admitted as being of the highest grade, says that he has carefully considered the opinions of others, and thoroughly observed the process in nature, and has been led to condemn support of the perineum as *irrational* and *useless* in *all* cases, and undoubtedly hurtful in some.

Having given you the means recommended by the various authors, I have plainly shown, I think, that we are at liberty to act as we best see fit, and in any case are re-enforced by authority. The earlier writers recommended merely the free use of lubricants and emollients, and from these simple means we can go even to the other extreme, and apply a fixed point d'appui, as recommended by Ramsbotham. Another point which all authors make, without exception, is that even with the means they advise, rupture will sometimes occur despite their act. Now, what is the only just conclusion to draw from this last statement? It is this: that where rupture occurs from the conformation of the female part, it will not be prevented by the means advised, if these means are applied to every case. If it is due to a too rapid labor or surprising of the perineum, it will not be prevented by the means advised, but the accoucheur should devote himself to retarding the labor.

If it be owing to the fact that the head is small, and does not undergo flexion, but on the contrary, is rapidly pushed through the parturient canal without assuming the proper position, the means indicated are undoubtedly to correct the position of the child's head. In this same manner I might review the long list of causes, as given by a number of writers, and suggest a different plan of prevention for each individual cause. This, then, should convince us that no one method is to answer in every case, and that the accoucheur who applies his means to suit the individual case, and who is adroit in detecting the danger, is the one in whose hands the lying-in woman will be most secure.

DISCUSSION.

Dr. Bartlett thought that a clear understanding of the causes which operate to produce rupture often indicate how we can avoid it. He considered the use of ergot a frequent one, and employed it with caution. In a modified way he favored support.

Dr. White said he wished to thank his young friend for bringing forward a subject so important. In the few minutes allotted to the discussion he could hardly do justice to the topic. Where the support was skillfully applied, he thought it could never do harm, and was often of the greatest value. Its object should be to keep the child's head in contact with its sternum, which is readily accomplished by passing the finger around the vertex, and at the same time pushing the chin upwards. The treatment, however, must of course vary somewhat in each instance. For many years he had been in the habit of making a slight incision down and outwards, on each side of the raphe, wherever a rupture was imminent. This is easily done with a pair of scissors, and if made during a pain, is hardly noticed by the patient. He did not remember having seen it resorted to until after he had practiced it himself. As a proof of the advantages of this method, an instance was cited of a woman forty-five years old, who went safely through an instrumental delivery after two lateral incisions had been made; and in another case, apparently better able to withstand the accident, but otherwise like the first, the perineum was ruptured extensively.

Dr. Mynter did not think the operation proposed by Dr. White was a new one. He had seen it made in Europe ten years before.

Dr. White said he had practiced the method fully twenty-five years.

Under voluntary communications, Dr. Mynter reported a curious case in which it was necessary to amputate the enlarged toe of a child, whose mother, when far advanced in pregnancy, had suffered from an ingrowing nail on the corresponding toe of her foot. After a brief discussion of the possible nervous connection between mother and child, the meeting adjourned.

